# Quantitative Systems Pharmacology Model for Therapies Targeting Aβ and Tau Pathologies in Alzheimer’s Disease

**DOI:** 10.1002/alz.089337

**Published:** 2025-01-09

**Authors:** Lin Lin, Sarah Minucci, Sarah DiBartolo, Joshuaine Grant, Kumar Kandadi Muralidharan, Matthew Hutchison, Jessica Collins, Amanda Edwards, Heike Hering, Thierry Bussière, Danielle Graham, Fei Hua, John Roberts

**Affiliations:** ^1^ Biogen, Cambridge, MA USA; ^2^ Applied BioMath, Concord, MA USA

## Abstract

**Background:**

Alzheimer’s disease (AD) is a progressive neurodegenerative disease characterized by the formation of amyloid‐beta (Aβ) plaques and neurofibrillary tangles (NFTs) composed of tau aggregates. Research in animal models has generated hypotheses on the underlying mechanisms of the interaction between Aβ and tau pathology. In support of this interaction, results from clinical trials have shown that treatment with anti‐Aβ monoclonal antibodies (mAbs) affects tau pathology. Meanwhile, preliminary results from a phase 1b study showed that BIIB080, a tau‐targeting antisense oligonucleotide (ASO), reduced tau biomarkers in early AD patients. Building upon our previous work of a quantitative systems pharmacology (QSP) model for anti‐Aβ mAbs, we have developed a more comprehensive model incorporating the interplay between Aβ and tau pathways and therapies targeting Aβ and tau.

**Method:**

A QSP model was developed that accounts for changes in Aβ and tau biomarkers in the progression of AD. It includes the production, aggregation, and inter‐compartment transport of both Aβ and tau species, as well as changes in phospho‐tau species and spreading of aggregated tau pathology. The model captures the interaction between Aβ and tau pathology by Aβ plaque‐dependent tau hyperphosphorylation that drives NFT formation in Braak region I/II and tau pathology spreading to Braak regions III/IV and V/VI. Furthermore, the model incorporates mechanisms of action for anti‐Aβ mAbs, including aducanumab, lecanemab, donanemab and gantenerumab, and the tau‐targeting ASO, BIIB080. The model was calibrated using Aβ and tau biomarker data (Aβ42/Aβ40, t‐tau and p‐tau181 in CSF and plasma, amyloid and tau PET) from natural history studies as well as pharmacokinetic (PK) data and biomarker response data from the clinical trials with these therapies.

**Result:**

Model simulations were in good agreement to the available clinical data. The calibrated model was able to match observed drug PK and Aβ and tau biomarker data, including amyloid re‐accumulation after depletion. In addition, the model was able to reasonably capture the effects of anti‐Aβ mAbs on tau pathology.

**Conclusion:**

A QSP model was developed to integrate therapies targeting Aβ and tau pathologies in AD. The model can be utilized to explore dosing regimens to support the development of combination therapy.

